# Is Fetal Endoscopic Tracheal Occlusion (FETO) a Predisposing Factor for Acid Gastro-Esophageal Reflux in Infants With Congenital Diaphragmatic Hernia?

**DOI:** 10.3389/fped.2020.00467

**Published:** 2020-08-25

**Authors:** Francesco Macchini, Anna Morandi, Stefano Mazzoleni, Martina Ichino, Giacomo Cavallaro, Genny Raffaeli, Carlo Ferrari, Silvana Gangi, Fabio Mosca, Isabella Fabietti, Nicola Persico, Ernesto Leva

**Affiliations:** ^1^Department of Pediatric Surgery, Fondazione IRCCS Ca' Granda Ospedale Maggiore Policlinico, Milan, Italy; ^2^NICU, Fondazione IRCCS Ca' Granda Ospedale Maggiore Policlinico, Milan, Italy; ^3^Department of Clinical Sciences and Community Health, University of Milan, Milan, Italy; ^4^Unit of Obstetrics, Fondazione IRCCS Ca' Granda Ospedale Maggiore Policlinico, Milan, Italy

**Keywords:** fetal therapy, congenital diaphragmatic hernia, gastro-esophageal reflux, pH-metry, neonatal surgery

## Abstract

**Introduction:** Various anatomical defects predispose patients with congenital diaphragmatic hernia (CDH) to develop gastroesophageal reflux disease (GERD). The fetal endoscopic tracheal occlusion (FETO) has increased the survival of patients with severe CDHs. The aim of this study was to study GERD in patients who underwent FETO.

**Materials and Methods:** We included patients with CDH treated with or without FETO (“FETO” and “no-FETO” group, respectively) from 2013 to 2016. Data on gestational age (GA), birth weight (BW), initial observed/expected lung to head ratio (O/E LHR), final O/E LHR, duration of ventilation and hospitalization, maximal tracheal diameter, and pulmonary volume were collected. All patients underwent pH-metry after 1 year of life, and the results were compared between groups and correlated to risk factors.

**Results:** Thirty-two patients were included in the study: 10 FETO and 22 no-FETO. No significant differences were observed in the pH-metric results of the two groups. No correlation was found between GA, BW, initial O/E LHR, maximal tracheal diameter, pulmonary volume, and pH-metric results. pH-metric results were correlated with the total duration of ventilation (*R* = 0.5, *p* = 0.003) and of hospitalization (*R* = 0.54, *p* = 0.001). Gastric herniation is associated with the worse pH-metric result.

**Conclusions:** The FETO procedure does not seem to represent an independent risk factor for GERD. However, patients with the most severe CDH have the worst GERD.

## Introduction

Congenital diaphragmatic hernia (CDH) is a rare malformation characterized by a defect in the diaphragmatic development allowing abdominal viscera to herniate in the chest (1, 2).

The degree of pulmonary hypoplasia and hypertension influences the survival and prognosis. Prenatal ultrasound allows stratifying fetuses with CDH into severe, moderate, and mild forms thanks to the observed/expected lung to head ratio (O/E LHR). The survival rate ranges between 0–20% and 70–100% (3).

Fetal endoscopic tracheal occlusion (FETO) has been reported to increase (from 24 to 49% in left-sided defects and from 17 to 42% for right-sided CDH) the postnatal survival of severe CDH compared to expectant management. This technique involves minimally invasive placement of an inflatable balloon into the fetal trachea under fetoscopy. Tracheal occlusion causes fluid accumulation into the fetal lungs, increases airway pressure causing cellular proliferation, and improves alveolar airspace and maturation of pulmonary vasculature (4, 5).

Among long-term comorbidities of CDH, gastroesophageal reflux disease (GERD) has been described with a prevalence of up to 81% of cases (6). Recently, some studies concerning severe consequences of GERD such as Barrett's esophagus and esophageal adenocarcinoma in CDH patients have been reported (7–9). In previous experiences from our center, a high frequency of GERD in a CDH series was recorded, and a routine assessment of GERD was suggested independently of symptoms for its potentially severe complications (10, 11).

Multiple mechanisms, both anatomical and functional, have been considered for GERD development in patients with CDH. Hence, several authors speculated that the worst diaphragmatic defects are associated with more severe GERD. Among extraintestinal predisposing factors for GERD in general population, the respiratory function has been demonstrated to play a role too (12, 13). What is actually known is that the FETO procedure leads to morphological changes of the trachea, thus conditioning the respiratory functions of these children in the 1st year of life (14–19).

Due to these considerations, this study was designed to evaluate if GERD is more severe in larger diaphragmatic defects and, in particular, if FETO may represent a predisposing factor to the development of higher-grade acid reflux as compared to patients without FETO.

## Materials and Methods

Medical records of all infants with CDH treated at our hospital between August 2013 and December 2016 were reviewed. The population was divided into two groups: the “FETO” group involving patients who underwent the FETO procedure, and the “no-FETO” group involving patients who did not receive the prenatal treatment.

The indication to the FETO procedure at our Fetal Surgery Center was based on the combined evaluation of the O/E LHR, measured by ultrasound (20), and the side of the defect (left, right, and bilateral). The FETO procedure was indicated for left CDH with O/E LHR ≤ 25% and for right CDH with O/E LHR ≤ 35% (21, 22), in the absence of major associated structural defects and chromosomal or genetic abnormalities. Fetal intervention was performed after parental informed consent and with approval by the local Institutional Review Board. The off-label use of the medical devices was approved by the Italian Ministry of Health.

The procedure was performed between 26 and 31 weeks of gestation, and the balloon (GOLDBALL 2 and COAX catheter; “*Balt Extrusion*,” Montmorency, France) was removed after 6 weeks (at around 34 weeks) usually by *in utero* puncture under endoscopic or ultrasound guidance (4).

After birth, the management of patients followed the international protocols of the European Congenital Diaphragmatic Hernia Consortium (23). When the patient was considered stable, surgical repair through median laparotomy was performed. The diaphragmatic defect was repaired directly, without prosthetic devices (primary repair) in grades A and B, according to the Congenital Diaphragmatic Hernia Study Group (CDHSG) Staging System (24); on the other hand, for C and D defects, a *Goretex*® patch was used.

In patients who required patch positioning, a chest drain was placed. A chest X-ray was performed 3 h after surgery and then according to the conditions of the newborn.

After hospital discharge, follow-up visits were scheduled in the thoracic outpatient office on a regular basis by a multidisciplinary team, including neonatologists, pediatricians, physiatrists, infant neuropsychiatrists, pneumologists, lung function technicians, pediatric surgeons, geneticists, audiologists, cardiologists, radiologists, and specialized nurses. In details, follow-up visits and plain chest X-rays were routinely performed at 1, 3, 6, 12, and 24 months even in asymptomatic children, mainly aimed to rule out recurrence of herniation and to evaluate lung development. Concerning GERD, as suggested by previous studies, all patients received oral proton pump inhibitors (PPIs) for the 1st year of life, specifically esomeprazole 0.8–1.2 mg/kg/day (25). Being part of our standardized protocols of follow-up for CDH, a 24-h pH-metry was performed on all children between the first and the 2nd year of life after suspending the PPI treatment at least 10 days before, independently of symptoms (11). PH-metry was always performed with a two-channel probe, with the proximal channel positioned at the junction of the mid- and lower third of the esophagus, 2.5 cm above lower esophageal sphincter, and the distal one in the stomach. During recording, infants' care was not altered, and oral feeding was continued. Parents were asked to register the occurrence of symptoms during the record. An episode of acid gastroesophageal reflux (GER) was defined when the esophageal pH fell below 4.0. Reflux was evaluated as the percentage of time of acid esophageal environment in the study period (RI, reflux index), the number of individual episodes, the number of events longer than 5 min, and the duration of the longest reflux. Furthermore, the severity of GERD was also determined in presence of interdigestive episodes of reflux, prolonged and/or nocturnal ones, and according to the symptom index (SI). On the basis of the studied pH-metric parameters, the tracings are classified as normal or pathological, in which case, patients are given antireflux medical therapy according to the current guidelines (26).

As previously mentioned, pH-metry is part of our standardized protocols of follow-up for CDH. We chose to perform pH-metry because it still remains a valid diagnostic tool to demonstrate acid in children with GERD, and the results of pH-metry seem easier to be compared between groups (27).

According to our internal protocol, the evaluation of GERD is completed with contrast X-ray and upper endoscopy with biopsies within the 6 to 8th year of life or before in presence of typical symptoms (10).

The pH-metry results were then compared between the FETO and the no-FETO group.

Among factors predisposing to GERD, gestational age (GA), birth weight (BW), initial and final O/E LHR, the increase in LHR pre- and post-FETO procedure (Δ-LHR), the total length of ventilation (invasive and not invasive), time of full enteral feeding, and the total length of hospital stay were derived from the patients' charts.

As physiopathology of GERD is strictly related to respiratory function (12, 13) and considering the fact that these results may be altered during the 1st year of life in CDH patients, the maximal tracheal caliber and the total lung volume at chest X-ray and the respiratory rate were collected at the time of pH-metry and were correlated to pH-metry itself.

Patients with infectious, neurological, cardiac, or metabolic disorders were excluded from the analysis. In addition, patients who underwent extracorporeal membrane oxygenation (ECMO), or other surgical thoracic or abdominal procedures, including prophylactic antireflux surgery, and patients with a recurrence of hernia were also excluded. This decision was taken to analyze a homogeneous population. Specifically, patients who received ECMO were excluded due to the limited number of patients (our ECMO program started in 2016) and to limit confounding factors related to the severity of the disorder and comorbidities.

PH-metric results were compared with the Student's *t*-test. Correlation between pH-metric results and predisposing factors was evaluated by Spearman correlation test. Statistical analysis was performed using SigmaStat® (Systat Software Inc., San Jose, CA, USA). A *p* < 0.05 was considered significant.

## Results

In the studied period, 32 patients met the inclusion criteria of the study: 10 in the FETO group and 22 in the no-FETO group. None of the children included in the study underwent thoracic or abdominal surgery after CDH repair. All patients were on full oral feeding at the time of evaluation.

Pre- and postnatal data, surgical characteristics, and clinical evaluations after 1 year of age of the two groups are reported in Table 1.

**Table 1:** Population data.

**Clinical features**	**FETO group**	**No-FETO group**	***p***
**(a) Clinical data**			
Patient no.	10	22	
Sex			
Males	5	14	0.7
Females	5	8	
Initial O/E LHR (%)	30.6 ± 5.6	51.3 ± 16.2	**0.002**
Final O/E LHR (%)	57.7 ± 12.1	49.4 ± 9.8	0.12
Δ O/E LHR (%)	27.1 ± 11.2	2.3 ± 11.0	**0.0001**
GA (weeks)	35.7 ± 2.5	38.2 ± 1.4	**0.001**
BW (grams)	2.600 ± 485	2.985 ± 700	0.13
**(b) Intra-operative data**			
Time at surgery (days)	3.3 ± 4.1	3.6 ± 3.4	0.83
Side of defect			
Left:	6	21	**0.02**
Right:	4	1	
Type of defect			
A+B:	4	17	0.056
C+D:	6	5	
Liver-up	8	4	**0.0015**
Stomach-up	5	9	0.712
Spleen-up	5	15	0.438
Sac	4	4	0.218
Diaphragmatic patch	6	5	0.056
**(c) Post-operative data**			
Length of invasive ventilation (days)	17.4 ± 5.5	12.2 ± 7.8	0.07
Length of non-invasive ventilation (days)	13.8 ± 6.7	8.3 ± 7.0	**0.04**
Time of full enteral feeding (days)	26.6 ± 15.9	22.5 ± 12.4	0.5
Length of hospital stay (days)	58.8 ± 23.4	43.2 ± 19.0	**0.05**
**(d) Follow-up data**			
Maximal tracheal caliber (mm)	10 ± 2.09	7.62 ± 1.18	**0.002**
Lung total volume (ml)	337 ± 46.7	446 ± 128.1	0.08
Respiratory rate (breaths/min)	33.9 ± 11.5	27.7 ± 4.3	0.08
GERD symptoms	5	4	0.096
Age at pH-metry (months)	20.9	16.5	0.21
Weight at pH-metry (kg)	9.2 ± 1	10 ± 1.3	0.13
Height at pH-metry (cm)	74.9 ± 4.8	76.6 ± 6.4	0.51

*The bold values help in highlighting statistically significant*.

In particular, no significant differences were recorded concerning sex distribution and birth weight, while GA was significantly lower in the FETO group. As regards to the prenatal data, the initial O/E LHR was significantly lower in the FETO group (mean 30.6 ± 5.6 vs. 51.3 ± 16.2, *p* = 0.002), and Δ O/E LHR was significantly higher in the FETO group (mean 27.1 ± 11.2 vs. 2.3 ± 11.0, *p* = 0.0001). The FETO group reached final O/E LHR similar to the no-FETO group.

Regarding surgical data, significant differences were limited to a higher incidence of right-sided hernia and liver herniation in the FETO group, while with the severity of the defect, the herniation of stomach and spleen, presence of hernia sac, and need for interposition of a patch were similar between the two groups.

Among postsurgical variables, a significant difference was observed only for the length of non-invasive ventilation (mean 13.8 ± 6.7 vs. 8.3 ± 7.0 days, *p* = 0.04) and of hospital stay (mean 58.8 ± 23.4 vs. 43.2 ± 19.0, *p* = 0.05), while the length of invasive ventilation and the time of full enteral feeding did not show differences.

Finally, at a median follow-up of 18 months (range 9–26 months), no significant differences were found in terms of total lung volume, respiratory rate, symptoms of GERD, and biometric data (age, weight, and height). Only the maximal tracheal caliber was significantly higher in the FETO group (mean 10 ± 2.09 vs. 7.62 ± 1.18 mm, *p* = 0.002).

As regards the comparison of the pH-metric recordings between the FETO and no-FETO groups, all the results are summarized in Table 2. No significant differences were recorded in the studied parameters. The age at the time of diagnostic evaluation was similar. The total number of acid refluxes in 24-h traces, the number of prolonged acid refluxes (>5 min), the length of the longest reflux, and the RI did not show significant differences.

**Table 2:** Comparison of pH-metric results between FETO and no-FETO group.

**PH-METRY**	**FETO**	**NO-FETO**	***p***
Age (months)	20.9	16.5	0.21
Reflux index (%)	4.95	3.6	0.48
Acid GER/24 h (num.)	69.5	72.23	0.88
Acid GER>5 min (num.)	1.04	2.3	0.23
Length of longest episode (minutes)	12.2	10.82	0.81
Pathological pH-metric tracts	5	9	0.71

The final classifications of the pH-metric tracts in normal or pathological also resulted the same between the two groups. Patients with pathologic findings were treated with medical therapy as the first line. All patients of our series were clinically negative at subsequent follow-up controls, and none required antireflux surgery in the first 2 years of life.

Considering the poor differences between the two groups, an analysis of the correlation among factors potentially predisposing to acid gastroesophageal reflux and the pH-metric recordings was carried on in all patients.

For continuous variables, the correlations were calculated with the Spearman test and are reported in Table 3. No correlations were found as regards to GA, BW, initial O/E LHR, Δ O/E, maximal tracheal caliber, lung volume, respiratory rate, and time of full enteral feeding. On the contrary, final O/E LHR was positively correlated to the RI (*R* = 0.53, *p* = 0.02). In addition, the length of ventilation (both invasive and non-invasive) and the length of hospital stay were positively correlated to the number of refluxes longer than 5 min.

**Table 3:** Correlation between potential predisposing factors and pH-metric recordings in general CDH population (Spearman test).

	**Reflux Index (RI) %**	**GER/24 h No**.	**GER>5^**′**^ No**.	**Longest GER**
GA	R:0.2	R:0.19	R:0.05	R:0.13
	p:0.26	p:0.3	p:0.8	p:0.48
BW	R:0.24	R:0.27	R:0.03	R:0.12
	p:0.18	p:0.13	p:0.88	p:0.51
Initial O/E LHR	R:0.2	R:0.23	R:0.19	R:0.04
	p:0.39	p:0.31	p:0.4	p:0.86
Final O/E LHR	**R:0.53**	R:0.24	R:0.42	*R:0.46*
	**p:0.02**	p:0.33	p:0.07	*p:0.048*
Δ-LHR pre/post-FETO	R:0.21	R:0.08	R:0.16	R:0.4
	p:0.5	p:0.83	p:0.69	p:0.4
Tot ventilation (inv + non-inv)	R:0.14	R:0.1	**R:0.5**	R:0.27
	p:0.43	p:0.58	**p:0.003**	p:0.13
Tot intubation	R:0.24	R:0.008	**R:0.54**	R:0.3
	p:0.18	p:0.96	**p:0.001**	p:0.09
Non-invasive ventilation	R:0.10	R:−0.13	**R:.46**	R:0.26
	p:0.56	p:0.48	**p:0.008**	p:0.15
Max tracheal caliber (>1 year)	R:0.28	R:0.34	R:0.14	R:0.16
	p:0.18	p:0.1	p:0.52	p:0.47
Lung volume (1 month)	R:0.19	R:0.03	R:0.27	*R:0.16*
	p:0.42	p:0.9	p:0.25	*p:0.5*
Respiratory rate	R:−0.08	R:0.26	R:−0.20	R:−0.26
	p:0.7	p:0.26	p:0.39	p:0.26
Length of hospital stay	R:0.19	R:0.1	**R:0.54**	R:0.3
	p:0.3	p:0.56	**p:0.001**	p:0.09
Time of full feeding	R:0.06	R:0.23	R:0.10	R:0.04
	p:0.76	p:0.23	p:0.60	p:0.85

*The bold values help in highlighting statistically significant*.

For categorical variables, the comparisons between potential predisposing factors and pH-metric recordings were calculated with the Student's *t*-test and are reported in Table 4. No significant differences in terms of severity of acid reflux were found as regards to sex, type and side of the diaphragmatic defect, presence of hernia sac, herniation of liver and spleen in thorax (liver-up/spleen-up), and the use of diaphragmatic patch. On the contrary, herniation in the thoracic cavity of the stomach (stomach-up) determined different pH-metric scores.

**Table 4:** Comparisons between potential predisposing factors and pH-metric recordings in general CDH population (*t*-test).

		**Reflux Index (RI) %**	**GER/24 h No**.	**GER>5^**′**^ No**.	**Longest GER**
Sex	M	3.8	66	1.5	9
	F	4.3	79	1.3	14
	*P*	0.8	0.42	0.83	0.42
Defect	A+B	3.5	72	1	11
	C+D	5	70	2.3	12
	*P*	0.44	0.9	0.2	0.85
Side	Left	4.4	74	17	12.5
	Right	1.9	56	0.2	4.5
	*P*	0.3	0.43	0.27	0.27
Sac	Yes	5	68	1.7	17.25
	No	3.7	72	1.3	9.25
	*P*	0.5	0.8	0.7	0.2
Stomach-up	Yes	6.2	84	3	18
	No	2.3	61	0.2	55
	*P*	**0.02**	0.16	**0.002**	**0.01**
Liver-up	Yes	5.6	78	2.6	14
	No	3	67	0.75	9
	*P*	0.15	0.5	0.06	0.34
Spleen-up	Yes	5.1	73	2.15	14
	No	2.45	69	0.25	6.5
	*P*	0.15	0.8	0.05	0.16
Patch	Yes	5	70	2.27	12
	No	3.5	73	1	10.5
	*P*	0.43	0.85	0.22	0.8

*The bold values help in highlighting statistically significant*.

Further analysis was done to assess the potential correlation between pH-metric data and variables significantly characterizing the FETO group. In detail, the O/E LHR pre-FETO was negatively correlated to the RI (*R* = −0.73, *p* = 0.02). No significant correlation was found for the other variables, including the GA, the herniation of liver, the length of non-invasive ventilation, and the maximal caliber of trachea. All these findings are reported in Table 5.

**Table 5:** Correlation between potential predisposing factors and pH-metric recordings in FETO population.

	**Reflux Index (RI) %**	**GER/24 h No**.	**GER>5^**′**^ No**.	**Longest GER**
GA	R:0.01	R:−0.31	R:0.02	R:0.25
	p:0.99	p:0.38	p:0.96	p:0.48
O/E LHR pre-FETO	**R:**–**0.73**	R:−0.57	R:−0.64	R:−0.49
	**p:0.02**	p:0.11	p:0.06	p:0.18
Non-invasive ventilation	R:0.09	R:0.16	R:0.43	R:0.15
	p:0.79	p:0.65	p:0.21	p:0.68
Tot intubation	R:0.42	R:0.37	R:0.48	R:0.39
	p:0.23	p:0.28	p:0.16	p:0.26
Max tracheal caliber (>1 year)	R:−0.04	R:−0.29	R:0.2	R:0.41
	p:0.94	p:0.53	p:0.67	p:0.35
Liver-up	Yes:5.31	Yes:72.87	Yes:2.5	Yes:11
	No:3.5	No:56	No:1.5	No:17
	p:0.76	p:0.62	p:0.77	p:0.59

*The bold values help in highlighting statistically significant*.

## Discussion

Because of the improvements in the neonatal care and surgical technique, the survival rate of CDH patients has markedly increased in recent years. Thus, the attention has been focused on medium- and long-term comorbidity. GERD has been previously demonstrated to represent a comorbidity of CDH, being characterized by a high frequency (up to 81% of survivors) and by potential severe complications, as Barrett's esophagus and esophageal carcinoma (8, 11). We recently demonstrated a surprisingly high prevalence of silent esophagitis in asymptomatic adolescents born with CDH (10). In CDH population, we also observed that acid GERD has similar features to that of other pediatric populations where the evaluation of GERD is considered mandatory, such as esophageal atresia. Prophylactic antireflux surgery at the time of CDH repair has also been proposed, but its requirement is debated for many reasons. First of all, for a technical issue, the procedure may determine a prolongation of the surgical time in newborns with limited general stability. Second, GERD presents a favorable course during life as part of this population. As regards to the other part of the population, unfortunately, the course of GERD is not so good, and medical therapy may need to be prolonged and antireflux surgery kept in consideration. Subsequently, considering also all predisposing factors for GERD and experiences showing a high incidence of silent esophagitis especially in severe CDH, we believe that studying the natural history of GERD in the 2nd year of life of patients treated for CDH is relevant for clinicians dedicated to their follow-up and may be helpful in preventing severe complications to patients. For these reasons, in our center, all CDH patients are treated with PPI within the 1st year of life, and a pH-metry is then performed in all patients after therapy interruption. This policy, on one hand, may overtreat some patients. On the other hand, considering the high incidence of silent GERD in CDH patients, its potential severe complications in the 1st year of life, and the absence of major side effects of PPI, we think that it may be justified in agreement with previous recommendations (11).

Multiple mechanisms, both anatomical and functional, have been hypothesized for GERD development in patients with CDH. The prenatal mediastinal shift and the compression of the intrathoracic esophagus may interfere with its development, causing ectasia and reducing the functionality of the lower esophageal sphincter (28). Asynchronies between thoracic and abdominal movements during breathing, recently demonstrated in CDH patients (29), may also contribute to lower esophageal sphincter dysfunction. In addition, intrathoracic dislodgement of the stomach associated with kinking and shortening of the gastroesophageal junction, alterations of the His angle, and diaphragmatic crura anomalies may contribute to GERD (30). The severity of the diaphragmatic defect has also been speculated to influence GERD (31). In addition, among extraintestinal factors predisposing to GERD, respiratory function has been demonstrated to play a role in the general population (12, 13).

In the last years, FETO procedure has been introduced as a prenatal minimally invasive procedure for CDH. It consists of the placement of an inflatable balloon into the fetal trachea under endoscopic guidance. Tracheal occlusion leads to fluid accumulation into the fetal lungs with the aim of increasing airway pressure into the bronchi, thus causing cellular proliferation and improving alveolar airspace. In addition, intrapulmonary fluids retained in the airways contain several growth factors needed for lung development. The procedure for severe CDH had shown an increase in the postnatal survival as compared to expectant management (3, 4). Currently, the results of only one small randomized controlled trial have been reported showing a significantly better survival in the FETO group (32). Further studies on the influence of FETO on survival, especially the so-called TOTAL trial, will better define this aspect (33). In our experience, the FETO procedure resulted effective in significantly improving the O/E LHR parameter. In fact, thanks to the FETO procedure, patients with CDH reached similar O/E LHR when compared to the no-FETO group. The improvement in the survival previously described by the literature was also confirmed in our series (3, 4). Thanks to this procedure, more severe CDH can currently survive. The population of survivors has to be considered as a new population of patients, having more severe defects that may potentially be more prone to develop early and late complications. In our experience, the FETO group had a lower gestational age, a higher prevalence of right-sided hernias, and liver herniation, thus justifying a higher need for long ventilation and hospital stay. Even if it is not significant in our series, larger defects (type C and D hernias) also seem to be more frequent in the FETO group with a consequent more frequent need for a patch to repair the diaphragmatic defects. The anatomical and functional consequences of the balloon placement on the patient trachea have been investigated. It is known that the FETO procedure leads to morphological changes of the trachea (14–16, 18, 19). In particular, it determines tracheomegaly. From a functional point of view, it seems that this tracheal dilation has an impact on the respiratory rate during the 1st year of life (17).

Due to all these anatomical and functional features, it may be speculated that GERD should be more severe in children treated with FETO as compared to those conservatively treated.

We, therefore, decided to compare the results of the routinary pH-metry within the 2nd year of life between the FETO and no-FETO groups.

As previously mentioned, pH-metry is part of our standardized protocols of follow-up for CDH. We chose to perform pH-metry because it still remains a valid diagnostic tool to demonstrate acid GERD in children, and the results of pH-metry seem easier to be compared between groups (27).

As reported in previous works (8–11) and in our experience, acid GERD presented a high prevalence in CDH patients, being on average 44%. This prevalence ranged from 41% in the no-FETO group to 50% in the FETO group. A recent study reported clinically relevant gastrointestinal complications in CDH patients after the FETO procedure, and its results are similar to our experience (34). As regards to the comparison between the two studied groups, no significant differences were found for all the evaluated parameters. The final diagnosis of our recordings (normal vs. pathological) confirmed the comparability of the two groups. On the basis of our findings, FETO does not seem to represent an independent risk factor for acid GERD. As opposed to our hypothesis, we can speculate that FETO, by promoting lung growth and contrasting the herniation of abdominal organs, might partially reduce the anatomical gastric distortion, thus limiting GERD.

Larger studies are advocated to confirm our observations, and the use of combined multichannel intraluminal impedance-pH monitoring may help in a better explanation of the physiology of GERD (acid and non-acid) in CDH patients.

Considering the absence of differences between the two groups, an analysis of the correlation among factors potentially predisposing to acid gastroesophageal reflux and the pH-metric recordings was carried on in the entire population.

Surprisingly, the majority of the potential factors, which have been previously described to influence GERD, did not show a significant correlation with the severity of acid reflux. Also, the anatomical characteristics of the diaphragmatic defect, such as type C and D defects, liver herniation, and patch repair, do not seem to be correlated with the severity of reflux. The only factor that significantly correlated with acid reflux was the herniation of the stomach. These observations may support the hypothesis that the intrathoracic dislodgement of the stomach, the kinking and shortening of the gastroesophageal junction, the reduction of the His angle, and the intrinsic esophagogastric dysmotility represent the principal etiopathogenetic factors for GERD in CDH children. Imaging and manometry are invoked to deepen this topic.

Among non-surgical variables in our population, only the total duration of ventilation (invasive and non-invasive) and of hospital stay positively correlated to the severity of acid refluxes. The exact underlying mechanism still has to be understood. However, we can speculate that prolonged intubation and use of sedation may interfere with antireflux mechanisms.

Considering the respiratory function, as reported in a previous work from our group, in our population, tracheomegaly was recorded after the FETO procedure, but in a long-term follow-up, it did not seem related to clinical complications (17). An etiopathogenetic correlation between GERD and respiratory problems in childhood has been exhaustively demonstrated in other populations. According to our results, tracheomegaly does not seem to determine worse GERD. Further studies with larger populations and more specific procedures are advocated. In addition, no correlations were observed between the severity of acid GERD and the respiratory variables, such as the total lung volume and the respiratory rate measured at the time of the evaluation. As the FETO group was characterized by worse values concerning the mentioned respiratory features, a further correlation was searched within this group. The only parameter that showed a negative correlation with the reflux index was the initial O/E LHR. Therefore, it seems that the degree of pulmonary hypoplasia may influence the severity of GERD. However, these data were not confirmed when analyzing the entire population, and the role of pulmonary hypoplasia on GERD still has to be investigated.

These findings support the feeling that the FETO procedure does not represent itself a potential risk factor for acid GERD.

The main limitation of our study is the limited number of patients and of the age range, so that further investigations are required to better understand the natural history of GERD in the FETO group (35). However, as far as we know, this is the first instrumental evaluation of the consequences of FETO for the gastrointestinal tract, and we believe that our results should act as a preliminary for the development of further studies.

## Conclusion

GERD confirms a high prevalence in our series of CDH patients. The FETO procedure does not seem to represent an independent predisposing factor for acid GERD. On the contrary, stomach herniation is related to worse results of pH monitoring. In addition, prolonged ventilation and hospitalization as well as the degree of lung hypoplasia seem to be related to worse acid GERD. We, therefore, recommend a strict follow-up of GERD in CDH patients, especially in the more severe ones.

## Data Availability Statement

The datasets generated for this study are available on request to the corresponding author.

## Ethics Statement

This study was approved by the Comitato Etico Milano Area 2, Fondazione IRCCS Ca' Granda Ospedale Maggiore Policlinico MIlano (Italy).

## Author Contributions

FMa, GC, EL, and FMo contributed conception and design of the study. IF and NP performed fetoscopy procedures and provided fetoscopic data. EL, FMa, and AM performed and helped in performing neonatal CDH repair surgical procedure. GC, GR, and SG performed NICU treatment and subsequent follow up. AM and SM organized the database. SM, FMa, AM, and MI performed the statistical analysis. FMa and MI wrote the first draft of the manuscript. AM and CF wrote sections of the manuscript. CF checked the final draft of the manuscript. All authors contributed to manuscript revision, read, and approved the submitted version.

## Conflict of Interest

The authors declare that the research was conducted in the absence of any commercial or financial relationships that could be construed as a potential conflict of interest.
